# FLAD1 is up-regulated in Gastric Cancer and is a potential prediction of prognosis

**DOI:** 10.7150/ijms.48162

**Published:** 2020-07-06

**Authors:** Pan Hu, Yuhang Pan, Chenyang Wang, Wenhui Zhang, He Huang, Jiani Wang, Nana Zhang

**Affiliations:** 1 Breast Cancer Center, the Third Affiliated Hospital of Sun Yat-sen University, Guangzhou, 510000, P.R. China.; 2 Department of Pathology, the Third Affiliated Hospital of Sun Yat-sen University, Guangzhou510000, P. R. China.; 3 Department of Urologic Oncology, Chongqing University Cancer Hospital & Chongqing Cancer Institute & Chongqing Cancer Hospital, Chongqing, 400030, P.R. China.; 4 Joint Surgery/Orthopedic Trauma Department, the Third Affiliated Hospital of Sun Yat-sen University, Guangzhou 510000, P. R. China.; 5 General Surgery Department, the Third Affiliated Hospital of Sun Yat-sen University, Guangzhou, 510000, P.R. China.

**Keywords:** FLAD1, biomarker, overexpression, gastric cancer, prognosis

## Abstract

**Background:** Gastric cancer (GC) is a common malignancy throughout the world. Biomarkers for prognosis and risk evaluation of GC are rapidly discovered. We investigated the prognostic role of FLAD1, an important protein-coding gene that affects cell cycle and survival.

**Methods:** The expression of FLAD1 at mRNA levels in GC tumor tissues and normal tissues was mined and analyzed in Oncomine database and verified in 10 pairs of GS tissues and their adjacent normal tissues in our center by RT qPCR. The FLAD1 protein expression were detected in 106 paraffin-embedded GC tissues by immunohistochemistry (IHC). Statistical analyses were applied to evaluate the clinical significance of FLAD1. The prognostic value of FLAD1 mRNA expression was also analyzed using the Kaplan-Meier plotter (www.kmplot.com).

**Results:** Statistics obtained from online database suggested FLAD1 mRNA was overexpressed in GC tissues. The results were further validated in 10 pairs of GS tissues and adjacent normal tissues in our center (p=0.021). IHC and survival analysis of GC samples from 106 patients showed FLAD1 was overexpressed in 63/106 (59.4%) patients and was associated to higher TNM stage (p=0.026). Multivariate analysis revealed FLAD1 was an independent prognostic factor for GC (p < 0.001). Furthermore, FLAD1 mRNA was associated to unfavorable overall survival (OS), first progression (FP), and post-progression survival (PPS) of GC (p<0.001).

**Conclusion:** FLAD1 in GC is overexpressed at both mRNA and protein level and could be a potential biomarker for GC prognosis.

## Introduction

Gastric cancer (GC) is the fifth most common and the third most mortal malignancy worldwide, and the second most common cause of morbidity and mortality in China^1^, ^2^. Most GCs are diagnosed at late stages when patients are complaining of abdominal pain, anorexia, and Cachexia. Consequently, the five-year survival rate of GC is dismal, despite great advances in surgery and adjuvant treatments 3. Hence, illuminating the molecular mechanisms of GC occurance and development is of great importance for improving clinical outcomes 4. Efforts are made to identify prognostic factors for better cost-effectiveness of GC management, such as coding and non-coding RNAs, which have been found to impact proliferation, apoptosis, and invasion of GC tumor cell 5, 6.

FLAD1 is a protein-coding gene for flavin adenine dinucleotide synthetase (FADS) ubiquitously expressed in various human tissue, with the highest level in lymph node 7. FADS is a key enzyme in the FAD biosynthesis process 8, which contains an N-terminal molybdopterin -binding (MPTb) domain and a C-terminal domain (FADS domain). MPTb domain has FAD hydrolase activity, and FADS domain catalyzes FAD synthesis 9. FADS is closely related to oxidation-reduction chain of cell. It participates in FMN biosynthesis, phosphor-adenosine phosphosulfate metabolism, and the oxidative-reduction process.

Human FLAD1 is ubiquitously expressed in lymph node, thyroid, and 25 other tissues 7. FLAD1 gene is located on the long arm of chromosome 1q21.3, the deficiency can cause multiple acyl-CoA dehydrogenase deficiencies (MADDs), which is a severe metabolic disorder with mitochondrial respiratory-chain deficiency 8, 10. FADS functions are critical to tumor cell as well. Due to its function in the oxidation-reduction chain, FLAD1 is strongly associated with the survival of malignant tumor cells and thus the prognosis of malignant tumor. Eeles et al. have found FLAD1, together with other 22 genes, was associated with prostate cancer 11. Target-identification phenotypic screening and competitive affinity-based proteome profiling have targeted FLAD1 as a potential target for treatment 12. Mitra et al. reported the relationship between FLAD1 expression and non-small cell lung cancer, especially in recurrent tumors, suggesting FLAD1 might be a biomarker for tumor relapse 13. Unfortunately, there is no study up to date investigating the relationship between FLAD1 and GC; whether FLAD1 can serve as a prognostic biomarker is unknown. To fill this gap, we mined the online database and employed our own sample library to validate the results. By investigating FLAD1 expression level in GC and its correlation to prognosis, we found FLAD1 was a valuable biomarker for the prognosis of GC.

## Methods and Materials

### Oncomine 4.5

To obtain the gene expression profile of *FLAD1* in GC tissue and normal tissue, we searched Oncomine (www.oncomine.org), an open-access online microarray database The data sets covered major types of cancer, including GC, and provided gene expression profiles based on more than 700 studies 14, 15. All data in our analysis were extracted in January 2020. The differences in FLAD1 expression between GC tissues, and normal gastric tissues were analyzed by Chi‑square test. The threshold value was determined as 2.0-fold change of expression level, p < 0.05, and top 10% gene rank. The details of 10 involved studies are as follows: 1. Diffuse Gastric Adenocarcinoma vs. Normal; p = 0.049, fold change = 1.537,5722 samples. Chen Gastric, Mol Biol Cell, 2003. 2. Gastric Intestinal Type Adenocarcinoma vs. Normal; p = 2.94E-11, fold change = 2.373, 343 samples. Chen Gastric, Mol Biol Cell, 2003. 3. Gastric Mixed Adenocarcinoma vs. Normal; p = 1.70E-4, fold change = 2.175, 971 samples. Chen Gastric, Mol Biol Cell, 2003. 4. Diffuse Gastric Adenocarcinoma vs. Normal; p = 0.001, fold change = 1.309,1088 samples. Cho Gastric, Clin Cancer Res, 2011. 5. Gastric Adenocarcinoma vs. Normal; p = 0.064, fold change = 1.440, 3202 samples. Cho Gastric, Clin Cancer Res, 2011. 6. Gastric Intestinal Type Adenocarcinoma vs. Normal; p = 0.056, fold change = 1.213, 5353 samples Cho Gastric, Clin Cancer Res, 2011. 7. Gastric Mixed Adenocarcinoma vs. Normal; p = 0.063, old change = 1.092, 5821 samples, Cho Gastric, Clin Cancer Res, 2011. 8. Gastric Cancer vs. Normal; p = 0.384, fold change = 1.047, 7615 samples. Cui Gastric, Nucleic Acids Res, 2011. 9. Gastric Intestinal Type Adenocarcinoma vs. Normal; p =3.92E-8, fold change = 1.440, 3202 samples. DErrico Gastric, Eur J Cancer, 2009. 10. Gastric Cancer vs. Normal; p = 0.003, fold change = 1.454, 865 samples.Wang Gastric, Med Oncol, 2010.

### Patients and Specimens

106 patients who were diagnosed with GC at the Third Affiliated Hospital of Sun Yat-sen University from Aug 2001 to Nov 2004 were included in the study. Among them 39 were male and 67 were female. The mean patient age at diagnosis was 57 (IQR: 43-68). The post-operative pathologic diagnoses confirmed gastric adenocarcinoma. None of the patients received neo-adjuvant chemotherapy. The clinicopathologic characteristics were evaluated according to the AJCC recommendation 16, 17 (Table 1). Follow-up time was defined from diagnosis to death or the lasted census date. Overall survival (OS) was defined from the date of first diagnosis to the date of death for any reason or to the last follow-up. The follow-up time of the GC cohort ranged from 1 to 118 months (median 21 months). We also collect of 10 pairs of GC tissues and their corresponding adjacent normal tissues in our center to investigate the different RNA and protein expression level of FLAD1 in GC and noncancerous tissues. The fresh tissue samples for Real-time PCR (RT-PCR) analysis were immersed into RNAlater (Sigma-Aldrich R0901, St. Louis., MO, USA) immediately during surgery and stored at 4˚C overnight, and then preserved at‑80˚C. The fresh tissue samples for western boltting analysis were preserved at ‑80 °C. Informed consent was obtained from all patients and the study procedure is approved by the ethical committee of the Third Affiliated Hospital of Sun Yat-sen University; Institutional Review Board (IRB) number, [2019] 02-071-01.

### Real-time PCR (RT-PCR) analysis

Total RNA samples were extracted from 10 pairs of GC tissues and their corresponding adjacent normal tissues using TRIzol reagent (Invitrogen, CA, USA). The extracted RNA was pretreated with RNase-free DNase, and 2 μg was used for cDNA synthesis. An initial amplification using *FLAD1* specific primers was performed for the PCR amplification of *FLAD1* cDNA, with denaturation at 95 °C for 10 min followed by 28 cycles of denaturation at 95 °C for 60 s, primer annealing at 58 °C for 30 s, and primer extension at 72 °C for 30 s. A final extension at 72 °C for 5 min was performed to complete of the cycles. The reaction mixture was then stored at 4 °C. Real-time PCR was performed to investigate the fold increase of *FLAD1* mRNA in each pairs of GC and normal gastric tissue using the following primer sequences designed by Primer Express v 2.0 software (Applied Biosystems): *FLAD1* fragments, 5'-TGACCCCTACTCCTGTAGCC-3' (forward) and 5'-AGCTGACGCAGAAAATCCCA-3' (reverse); and GAPDH, 5'-TGTTGCCATCAATGACCCC-3' (forward), 5'-CTCCACGACGTACTCAGC-3' (reverse). Glyceraldehyde-3-phosphate dehydrogenase (GAPDH) was used as an internal control; the relative expression level of FLAD 1 was calculated using the 2-ΔΔCT method. All experiments were performed in triplicate.

### Western blotting analysis

Cells at 70‑80% confluency were lysed by radioimmunoprecipitation assay buffer (RIPA; Cell Signaling Technology, Inc., Danvers, MA) with complete protease inhibitor cocktail (Roche Applied Science, Mannheim, Germany) on ice. Fresh 6 pairs of GC tissues and their corresponding adjacent normal tissues were ground into powder in liquid nitrogen and then lysed using SDS-PAGE sample buffer. A total of 20 μg samples were separated on 10.5% SDS polyacrylamide gels and transferred onto PVDF membranes (Immobilon P, Millipore, MA). PVDF membranes were blocked with 5% fat-free milk in Tris-buffered saline with 0.1% Tween-20 at room temperature for 1 h, and then incubated with anti-FLAD1 antibody (Abnova Corp., TW, catalog Number PAB22183) at 1:500 dilution overnight at 4°C, and then incubated with horseradish peroxidase-conjugated goat anti-rabbit IgG (Santa Cruz Biotechnology, SC-2004). FLAD1 expression was detected by ECL Western blotting detection reagent (Amersham) according to the manufacturer. GAPDH was used as loading control.

### IHC Staining and Analysis

The paraffin-embedded samples of 106 human gastric cancer in the Third Affiliated Hospital of Sun Yat-sen University from Aug 2001 to Nov 2004 were obtained to perform IHC staining. Each 4-μm-thick paraffin slide was treated with xylene and rehydrated with alcohol solution in a descending concentration. The slides were then treated with EDTA antigenic retrieval buffer in microwave at 650W for 3min and then 350W twice more. Hydrogen peroxide (3%, in methanol) was used to suppress the endogenous peroxidase activity. The samples were incubated with 1% BSA at room temperature for 60min to block unspecific binding, and then incubated with rabbit polyclonal antibody raised against recombinant FLAD1 (Abnova Corp., TW, catalog Number PAB22183), 4 °C at 1:500 dilution level overnight. Common goat serum (Santa Cruz Biotechnology, Inc.) was used as the negative control. After 3 washes in PBS, the samples were incubated with anti-rabbit secondary antibody at room temperature for 30min, and further incubated with a streptavidin-horseradish peroxide complex (1:1500, Abcam, Cambridge, UK) at room temperature for 30min. After incubation, the slides were stained with 3-amino-9-ethyl carbazole for 3 min at room temperature and then counterstained with 10% Mayer's hematoxylin for 30s. The samples were then dehydrated for analysis.

The immunostaining was analyzed by two pathologists blinded to patient-related information. The extent of IHC staining was categorized into 0-no staining (no visible difference from control group), 1-weak staining (light yellow), 2-moderate staining (yellow), and 3-strong staining (dark yellow). The proportion of tumor cell in the sample was scored as 0-no positive cells, 1-positive cells consists 1~25% of the sample, 2-positive cells consists 26~50% of the sample, 3-positive cells consists 51~75% of the sample, and 4-positive cells consists 76~100% of the sample. The FLAD1 expression was evaluated by the extent score multiplied by the proportion score. Total score 0: negative (-); total score 1~4: weakly positive (+); total score 6~8: positive (++); and total score 9~12: strongly positive (+++).The chosen of cut-off values for FLAD1 was based on the heterogeneity using log-rank test concerning overall survival. A staining score of ≥8 was used to define high FLAD1 expression, and <8 was used to define low FLAD1 expression.

### Statistical Analyses

The IHC results and corresponding clinicopathologic features were analyzed by SPSS Statistics 23 (IBM Corp., NY, US). The correlation between FLAD1 expression and other clinical features (age, sex, grade, TNM stage, and tumor infiltration) was analyzed using the χ^2^ test and Fisher's exact test while *n* < 40. Cox-regression model was used to analyze the impact of FLAD1 and covariates on survival. The impact of FLAD1 expression overall survival was validated by our 106 cases using the Kaplan-Meier plot, and evaluated by log-rank p-value and HR.

### The Kaplan-Meier plotter

The Kaplan-Meier plotter (www.kmplot.com) is an online graphic tool to explore the relationship between gene expression and prognosis. Using the website's built-in data, the Kaplan-Meier plotter can provide survival curves according to different gene expression level. It also provides utilities including subgroup analyses based on several clinicopathological parameters (clinical staging, receptor status, etc.) 18. The cutoff value of gene expression level was determined automatically by selecting the “auto select best cutoff” option. The survival curves of the two cohorts were plotted to evaluate OS, FP, and PPS. To quantify the impact of FLAD1 overexpression on prognosis, the number at risk, log-rank p-value and hazard ratio (HR) with 95% confidence interval (CI) were calculated and annotated for each plot.

## Results

### FLAD1 was overexpressed in GC

**Figure 1:**
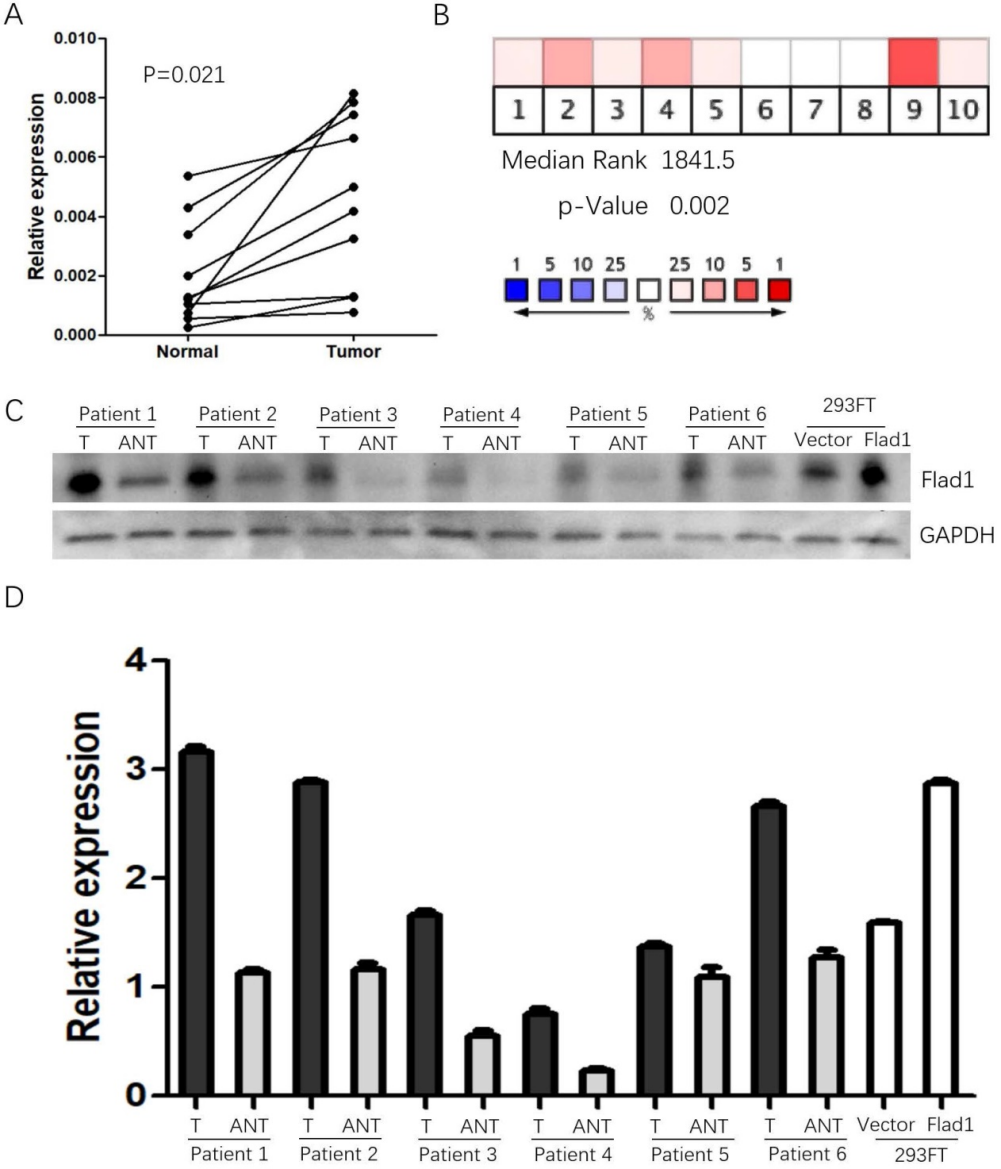
Expression of FLAD1 at mRNA level in GC and non-cancerous tissue. **A.** Heat map revealed over-expression of FLAD1 in GC across 10 studies. **B.** Expression levels of FLAD1 in 10 paired (normal vs tumor) GC samples were detected by real-time PCR. C. FLAD1 protein expression levels in six paired gastric carcinoma tissues and over expression of FLAD1 in 293FT cells by Western blotting. D. Quantitative analysis of FLAD1 protein in **C.** T, Gastric carcinoma tissues, ANT, matched adjacent non-tumor gastric tissues.

The data obtained from Oncomine suggested elevated *FLAD1* expression in GC compared to normal tissues (median rank: 1841.5.0, *p* =0.002, Figure 1A). As a validation of the results, the qPCR analysis of 10 paired (normal vs GC) samples from our patient cohort was performed (Figure 1B). Consistent with these data, FLAD1 protein was also found to be upregulated in 6 fresh GC tissues compared with non-cancerous tissues (Figure 1C, D).

### FLAD1 overexpression was associated with poor outcome of GC

**Figure 2:**
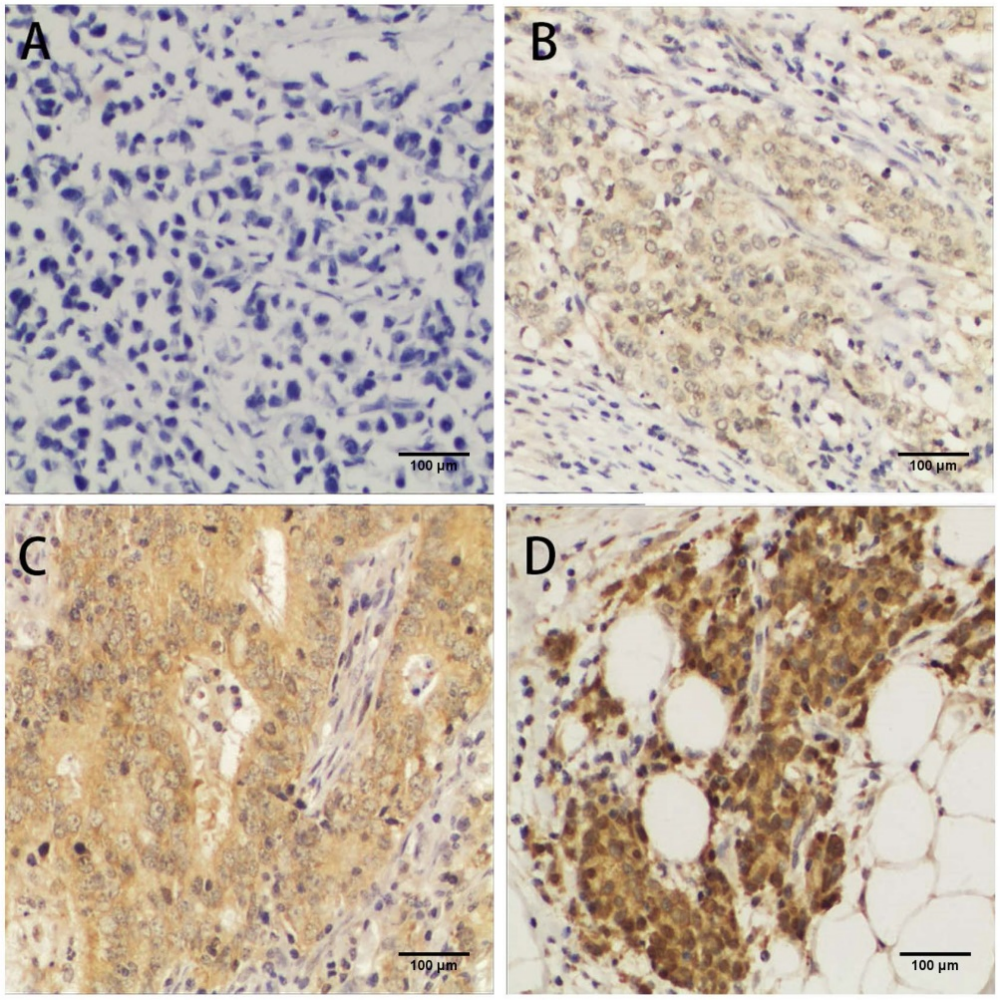
Immunochemistry analyses of FLAD1 expression in GC tissue samples. FLAD1 expression was mainly localized in cytoplasm. Representative images of **A.** Negative staining of FLAD1, **B.** weakly positive staining (+) of FLAD1, **C.** positive staining (++) of FLAD1, and **D.** strongly positive staining (+++) of FLAD1. The magnification was 400×.

To investigate the correlation between FLAD1 overexpression at protein level and prognosis of GC, we performed IHC analysis based on the 106 patient samples. The extent of staining was scored into five levels compared to non-cancerous tissue (Figure 2). The staining for FLAD1 was positive for 63/106 (59.4%) patients. The sex, tumor size, tumor grade, metastasis, infiltration status and Ki-67 expression level were not of significant difference between the FLAD1-positive and the FLAD1-negative group (Table 1), though the majority of patients had pT3 disease (79.2%). FLAD1 overexpression was correlated to patient age ≥ 60 years (Pearson χ^2^ = 5.014, p = 0.024), higher pathologic T stage (Pearson χ^2^ = 25.358, p < 0.001), higher pathologic N stage (Pearson χ^2^ = 33.247, p < 0.001), and higher TNM stage (Pearson χ^2^ = 9.288, p = 0.026). The correlation was further supported by non-parametric testing (Table 2).

**Figure 3:**
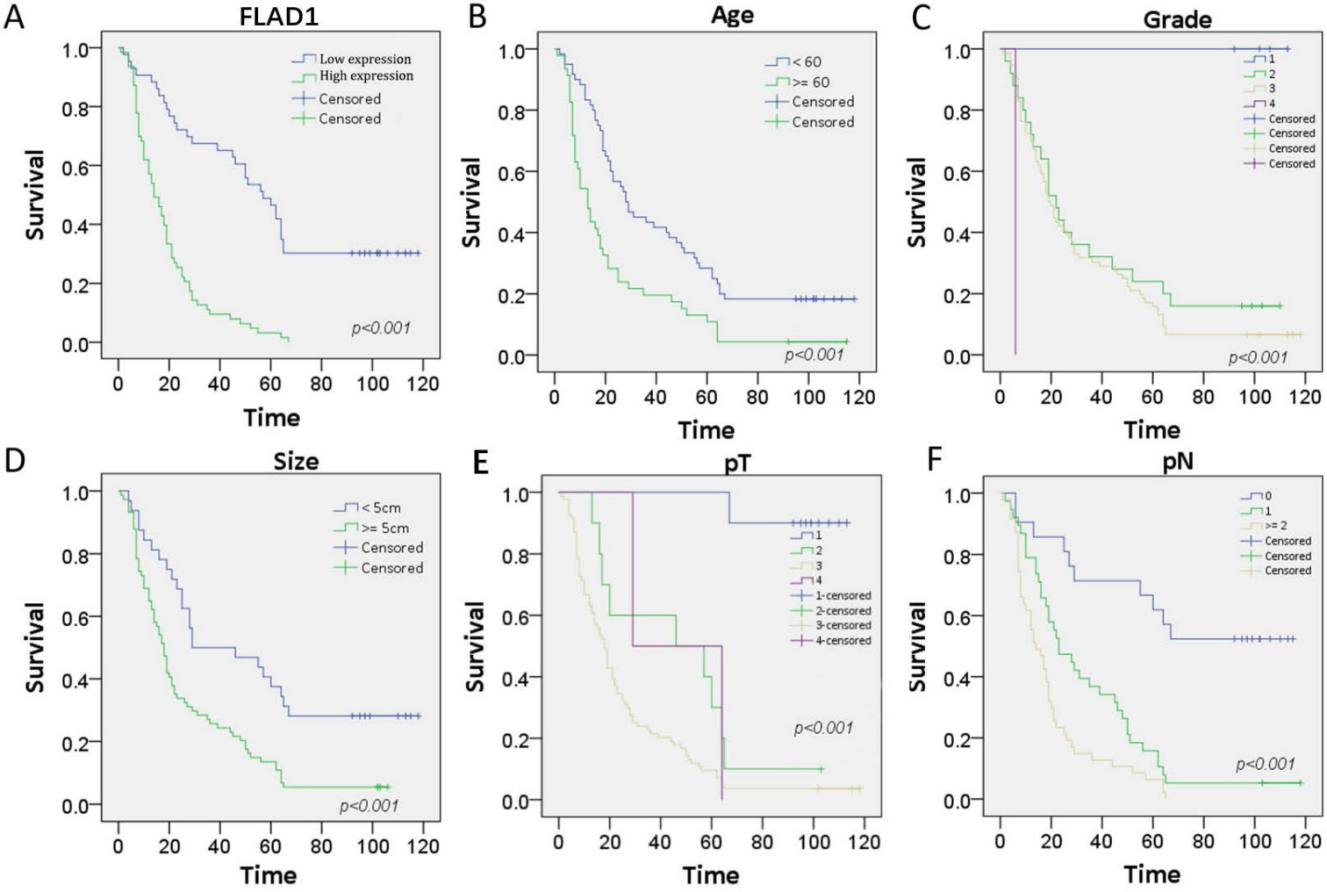
Kaplan-Meier survival curves with univariate analysis (log-rank test) of the 106-patient cohorts confirmed FLAD1-positive individuals had worse OS. Patient age, tumor grade, tumor size, and TNM stage also had impact on the OS of GC. **A.**OS rate for GC patients with high FLAD1 expression compares to those with low FLAD1 expression. **B.** OS rate for GC patients with age over 60 years compares to those with age under 60 years. C.OS rate for GC patients with grade 1, grade 2, grade 3, and grade 4. **D.** OS rate for GC patients with tumor size over 5cm and under 5cm. **E.** OS rate for GC patients with pT1(Tumor invades lamina propria, muscularis mucosae, or submucosa), pT2 (Tumor invades muscularis propria), pT3(Tumor penetrates subserosal connective tissue without invasion of visceral peritoneum or adjacent structures), and pT4(Tumor invades serosa (visceral peritoneum) or adjacent structures). **F.** OS rate for GC patients with pN0 (No regional lymph node metastasis), pN1(Metastasis in 1-2 regional lymph nodes), and pN2 (Metastasis in 3-6 regional lymph nodes).

The prognostic role of FLAD1 was further validated by the patient cohorts in our center using Cox regression model. The median follow-up time for this cohort is 21 months. On univariate analysis, we confirmed FLAD1-positive individuals had higher risk of death (HR = 4.388 p < 0.001). Meanwhile, patient age ≥ 60 years, tumor size ≥ 5cm, and higher TNM stage were also death-related risk factors. On multivariate analysis, our results supported FLAD1 (HR = 2.937, p < 0.001) was an independent risk factor for OS (Table 3). The Kaplan-Meier plots revealed FLAD1-positive GC patients had worse OS compared to FLAD1-negative ones (Log-rank χ^2^ = 41.978, p < 0.001, Figure 3A). Elder age, higher grade, greater tumor size, higher pT and pN stage also indicated poor outcomes (Figure 3B-F).

**Figure 4:**
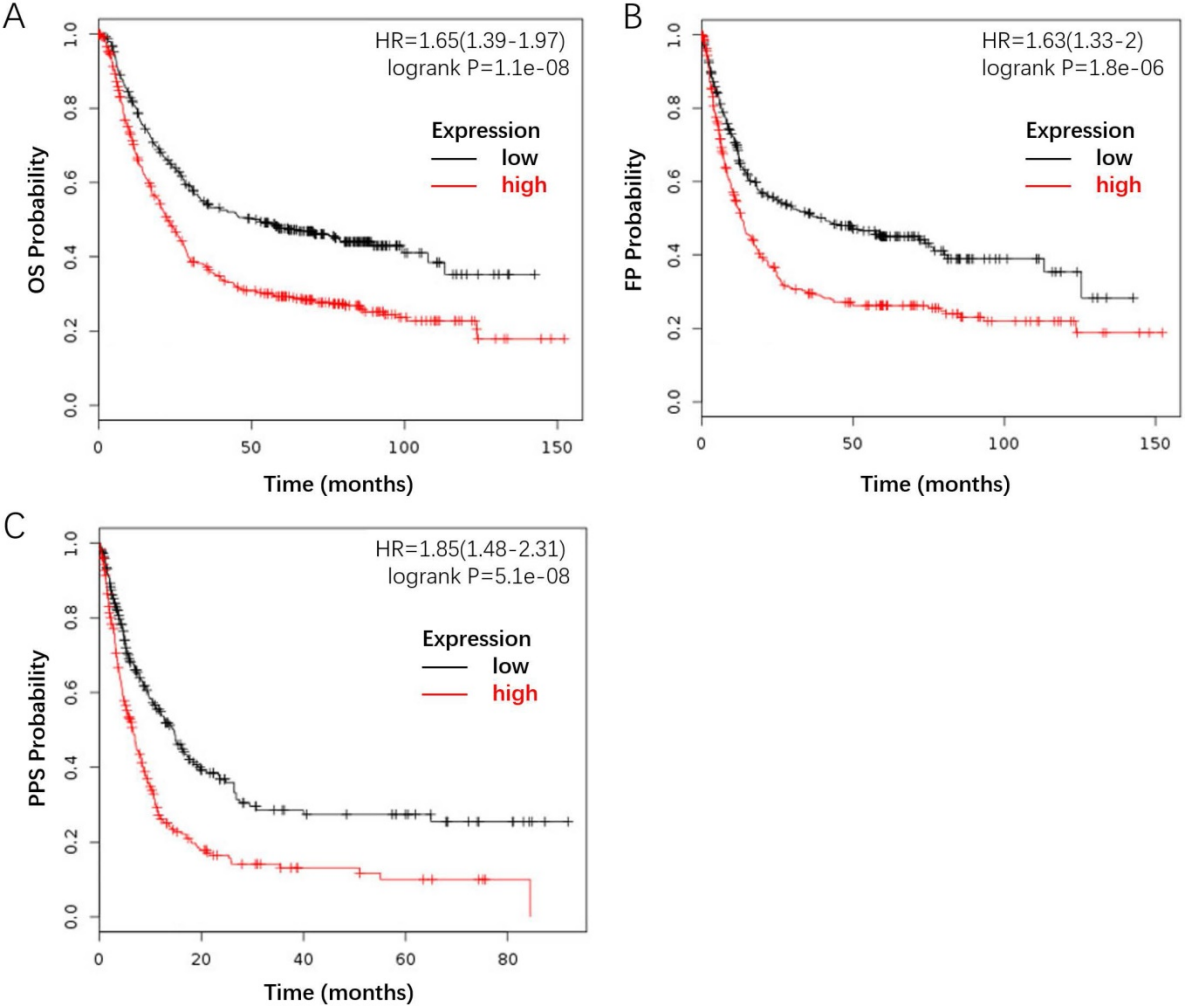
*FLAD1*-overexpressing individuals had a worse outcome compared to the baseline. The survival plot was generated by the Kaplan-Meier Plotter using the website's built-in dataset. **A.** OS rate for GC patients with high FLAD1 expression (480 patients) compares to those with low FLAD1 expression (396 patients). **B.** FP rate for GC patients with high FLAD1 expression (327 patients) compares to those with low FLAD1 expression (314 patients). **C.**PPS rate for GC patients with high FLAD1 expression (248 patients) compares to those with low FLAD1 expression (251 patients).

Furthermore, we investigated the correlation between *FLAD1* overexpression at mRNA level and prognosis of GC by plotting and comparing the OS, FP, and PPS of GC patients to healthy individuals through Kaplan-Meier plotter (www.kmplot.com). *FLAD1* overexpression was associated with worse OS (HR = 1.65, 95% CI: 1.39-1.97, p < 0.001), FP (HR = 1.63, 95% CI: 1.33-2.00, p < 0.001), and PPS (HR = 1.85, 95% CI: 1.48-2.31, p < 0.001, Figure 4A-C). Taken together, these findings supported that *FLAD1* was a satisfactory prognostic factor for GC patients.

## Discussion

GC is the second leading cause of cancer-related death worldwide 19. Current treatment for GC includes surgery, chemotherapy, adjuvant/neoadjuvant chemotherapy, and radiotherapy 20-23. The alternatives of treatment necessitate accurate prediction of tumor-related risks. The TNM-stage-based classification proposed by the AJCC was the most adopted evaluation for GC 24; meanwhile, histopathologic grading has been reported to be an independent prognostic factor, with several related grading system under construction 25. Clinicopathologic characteristics such as cellular dysmorphism, tumor location, comorbidity, and complications are also involved in some monograms 26. However, these prognostic factors have some limitations. First, despite satisfactory accuracy and prognostic power, the traditional TNM staging system is largely based on surgery, which is not regular in non-operable patients. Secondly, the TNM staging system cannot well adapt to the fact that the anatomic location of the primary tumor is influential to prognosis 24. Thirdly, the prognostic power of clinical features is often refined to specific stages; besides, there is no unanimous agreement on the threshold value and criteria for these factors. As a response to these challenges, tumor biomarkers are increasingly evaluated for the prognosis of GC 27-29.

FLAD1 is an essential gene in flavin metabolism and oxidative-reduction chain. It is expressed across more than 200 species. In the human genome, FLAD1 is located on chromosome 1 (1q21.3) and most highly expressed in lymph nodes, although ubiquitously expressed in all tissue types 7. FLAD1's protein product, FADS, possesses basic yet indispensable oxidative-reduction bioactivity. For this reason, it is widely distributed in the cytoplasm and membranous organelles such as mitochondrion 30. More importantly, due to the significant role in electron transportation, it is closely correlated to cell metabolism. FLAD1 has already been identified to correlate with susceptibility and outcome of hepatocellular carcinoma, prostate cancer and lung cancer 11, 13, 31. To our knowledge, there is no current study investigating the prognostic role of FLAD1 in GC. Our study first confirmed a correlation between FLAD1 overexpression and poor outcome of GC.

The present study verified that FLAD1 mRNA was overexpressed in GC tissues compared with normal matched tissues staining which provided novel evidence that the up-regulation of FLAD1 was closely associated with poor survival rates in GC patients. Multivariate analysis revealed FLAD1 was an independent prognostic factor for OS in patients with GC. To investigate the clinical significance of FLAD1 expression at mRNA level, we analyzed its relationship with prognosis of GC by plotting and comparing the OS, FP, and PPS of GC patients to healthy individuals by using The Kaplan-Meier plotter (www.kmplot.com) and found that *FLAD1* mRNA was associated to unfavorable OS, FP, and PPS of GC (p<0.001). Taken together, these findings clearly demonstrated that FLAD1 was a satisfactory prognostic factor for GC patients. It may serve as a biomarker for GC, which may aid early diagnosis and precise therapy. However, at present, the precise functions of FLAD1in malignant cancer remain obscure. Probing the precise mechanism underlying FLAD1 in GC requires further investigation.

We additionally investigated the association between FLAD1 expression and other clinical features of patients with GC. FLAD1 overexpression was correlated to patient age ≥60 years, higher pathologic T stage, higher pathologic N stage, and higher TNM stage, which revealed that FLAD1 may serve as a molecular biomarker for a subpopulation of patients with more aggressive disease. However, the sex, tumor size, tumor grade, metastasis, and infiltration status were not of significant difference between the FLAD1-positive and the FLAD1-negative group.

To testify whether *FLAD1* is a satisfactory prognostic factor for GC, we used the Kaplan-Meier plotter to analyze the correlation between survival and *FLAD1* expression level. The OS, FP, and PPS were all reduced in *FLAD1*-overexpressing groups, after a follow-up time of 150 months (OS/FP) and 80 months (PPS), respectively.

The survival analyses for different clinical stages, lymph node metastasis status, and HER2 status were also conducted. Recently researchers have started to investigate the association between FLAD1 and cancer. However, the exact role of FLAD 1 in human cancers does not appear to be clearly identified. Another study of our team in breast cancer has found the expression of FLAD 1 is closely associated with genes regulating DNA replication, microtubule, mitosis, cytoskeleton, cell cycle, cell division, p53 signaling pathway, nucleotide excision repair, and mismatch repair (data not showed). Based on these relationships, it could be hypothesized that FLAD1 may participated in cancer cell proliferation, migration and DNA damage repair. One limitation of this study was short of research on molecular mechanism. It remains ambiguous how the FLAD1 exerts unique impact on cancer development. Nevertheless, FLAD1 expression still offers a path forward towards a future GC risk assessment system.

In conclusion, to the best of our knowledge, we first reported FLAD1 was overexpressed and associated with poor prognosis of GC. Current understanding of the mechanism underlying our findings is lacking; the paucity of research on the pathway involved in FLAD1 overexpression also limits further development of its therapeutic application. Future researches are needed to investigate the causality and molecular mechanism of FLAD1 overexpression. Nevertheless, our study still highlights FLAD1 as a potential prognostic factor for GC in the clinical context. Its expression status may serve for the early diagnosis and stratification of patients and create a new therapeutic selection for GC patients.

## Figures and Tables

**Table 1:** Association between FLAD1 expression level and clinicopathological characteristics

Patient characteristics	FLAD1 expression		Total (%)	P-value
	Low (%)	High (%)		
**Sex**				0.629
Male	17 (16.0)	22 (20.8)	39 (36.8)	
Female	26 (24.5)	41 (38.7)	67 (63.2)	
**Age**				0.024
< 60 years	30 (28.3)	30 (28.3)	60 (56.6)	
≥60 years	13 (12.3)	33 (31.1)	46 (43.4)	
**Size**				0.083
< 5cm	17 (16.0)	15 (14.2)	32 (30.2)	
≥5cm	26 (24.5)	48 (44.0)	74 (69.8)	
**pT**				< 0.001
1	9 (8.5)	1 (0.9)	10 (9.4)	
2	9 (8.5)	1 (0.9)	10 (9.4)	
3	24 (22.6)	60 (56.6)	84 (79.2)	
4	1 (0.9)	1 (0.9)	2 (1.9)	
**pN**				< 0.001
0	16 (15.1)	5 (4.7)	21 (19.8)	
1	22 (20.8)	16 (15.1)	38 (35.8)	
≥2	5 (4.7)	42 (39.6)	47 (44.3)	
**Metastatic disease**				1.000
Yes	3 (2.8)	4 (3.8)	7 (6.6)	
No	40 (37.7)	59 (55.7)	99 (93.4)	
**TNM stage**				0.026
I	7 (6.6)	6 (5.7)	13 (12.3)	
II	11 (10.4)	7 (6.6)	18 (17.0)	
III	25 (23.6)	43 (40.6)	68 (64.2)	
IV	0 (0)	7 (6.6)	7 (6.6)	
**Pathologic grade**				0.321
Low	14 (13.2)	15 (14.2)	29 (27.4)	
High	29 (27.4)	48 (45.3)	77 (72.6)	
**Infiltration**				0.646
Yes	3 (2.8)	2 (1.9)	5 (4.7)	
No	40 (37.7)	61 (57.5)	101 (95.3)	
Ki67 expression				
Negative	16 (40.0)	24 (60.0)	40 (37.8)	0.926
Positive	27 (40.9)	39 (59.1)	66 (62.2)	

**Table 2:** Pearson χ^2^ and Spearman rank-sum correlation coefficient of clinicopathologic characteristics associated with FLAD1 overexpression

Clinicopathologic features	Pearson χ^2^	Correlation coefficient
Age	5.104	0.219
pT	25.358	0.449
pN	33.247	0.555
TNM stage	9.288	0.270

**Table 3:** Cox-regression analysis of various prognostic parameters in GC patients

Risk factor	Univariate		Multivariate	
	HR (95% CI)	P-value	HR (95% CI)	P-value
Age ≥60	2.077 (1.375-3.139)	0.001	1.533 (0.978-2.404)	0.063
Size ≥5 cm	2.277 (1.410-3.678)	0.001	1.176 (0.690-2.001)	0.551
**pT**		0.001		0.005
1	Referent		Referent	
2	17.539 (2.207‑139.398)	0.007	16.415 (1.870‑144.062)	0.012
3	36.233 (4.970‑264.173)	< 0.001	23.874 (2.760-206.479)	0.004
4	16.855 (1.516‑188.064)	0.022	13.411 (0.973‑184.922)	0.052
**pN**		< 0.001		0.577
0	Referent		Referent	
1	4.022 (1.955‑8.274)	< 0.001	1.212 (0.525‑2.798)	0.652
≥2	7.015 (3.421‑14.386)	< 0.001	1.511 (0.641-3.514)	0.346
**TNM stage**		0.013		0.051
I	Referent			
II	2.015 (0.866-4.688)	0.104	2.891 (1.121-7.457)	0.028
III	2.257 (1.076-4.736)	0.031	3.910 (1.397-7.287)	0.006
IV	2.665 (0.963-7.380)	0.059	3.194 (1.007-9.470)	0.036
FLAD1 expression	4.388 (2.715-7.092)	< 0.001	2.937 (1.522-5.760)	0.001
